# A pharmacovigilance study of association between proton pump inhibitor and dementia event based on FDA adverse event reporting system data

**DOI:** 10.1038/s41598-021-90108-7

**Published:** 2021-05-21

**Authors:** Bin Wu, Qiaozhi Hu, Fangyuan Tian, Fengbo Wu, Yuwen Li, Ting Xu

**Affiliations:** 1 Department of Pharmacy, West China Hospital, Sichuan University, 37 Guoxue Alley, Wuhou, Chengdu, 610041 Sichuan China; 2 West China School of Pharmacy, Sichuan University, Chengdu, 610041 Sichuan China

**Keywords:** Dementia, Drug safety

## Abstract

Proton pump inhibitor (PPI) was widely used around the world. Studies suggested conflicting results between PPI treatment and dementia event. This study examined the association between six PPI agents and dementia event by mining the US FDA Adverse Event Reporting System (FAERS) database from 2004 to 2020. We employed proportional reporting ratio (PRR) and information element (IC) methods to detect the signals of dementia relevant to PPI. We also analyzed characteristics of PPI and positive control reports, compared dementia event between long- and short-duration of PPI treatment. Finally, we identified 2396 dementia cases with PPI treatment. We did not detect significant signal between PPI and dementia event: PRR = 0.98, 95%CI 0.94 to 1.02, IC = −0.03, 95%CI − 0.17 to 0.10, even in gastroesophageal reflux disease cases: PRR = 0.65, 95%CI 0.59 to 0.72, IC = −0.62, 95%CI − 0.97 to − 0.27. No significant differences of dementia event were detected between long- and short- duration groups, the OR (95%CI) of the 3 years, 5 years and 10 years comparison were 0.70 (0.48 to 1.02), 0.72 (0.45 to 1.15) and 1.65 (0.75 to 3.63), respectively. Based on the current FAERS data mining, we discovered no association between PPI use and dementia event, even in long-term PPI therapy case.

## Introduction

Proton pump inhibitor (PPI) was commonly used worldwide, to treat peptic ulcer disease (PUD), gastroesophageal reflux disease (GERD), *Helicobacter pylori* infection, or prevent side effects of glucocorticoids or non-steroidal anti-inflammatory drugs (NSAIDs)^1^. However, PPI agents were also overused by off label indication, excessive dosage and long-term treatment^2,3^.

With the widespread use of PPI agents, numerous studies concerned the safety of PPI treatment^4–7^. The association between PPI therapy and dementia event was a hot issue. PPI agents were reported to increase β-amyloid (Aβ) levels in the mouse brain by affecting the β- and γ-secretase enzymes^8^, and to lead to vitamin B12 deficiency which was associated with cognitive impairment^9^. Some studies reported PPI use could increase dementia event^10–16^. More recent studies found no significant association between PPI and dementia^17–21^. Professor Lai et al. expounded, to test dementia event, the potentially offending agent should be taken for a long time, such as PPI in GERD treatment^22^. However, the association between long-term PPI use and dementia event was also conflicting^23–25^.

Adverse event reporting system (AERS) data was an outstanding source for pharmacovigilance analysis and post-marketing drug safety monitoring. The United States Food and Drug Administration AERS (FAERS) is one of the biggest databases open to the public^26^. To the end of 2020, FAERS had gathered more than twelve millions of adverse cases reported by both health professionals and non-health professionals. The FAERS data could be used to detect signals of drug-associated adverse event by data mining methods^27–29^. Data mining was shown to be effective in continuous pharmacovigilance monitoring of drug safety issues for old drugs such as PPI^30^. To the best of our knowledge, there was no research concerning the association between PPI and dementia based on FAERS database. The objective of present study was to detect the association between PPI use and dementia event by comprehensively assessing spontaneous reports submitted to the FAERS database.

## Results

### Characteristics analysis

**Figure 1:**
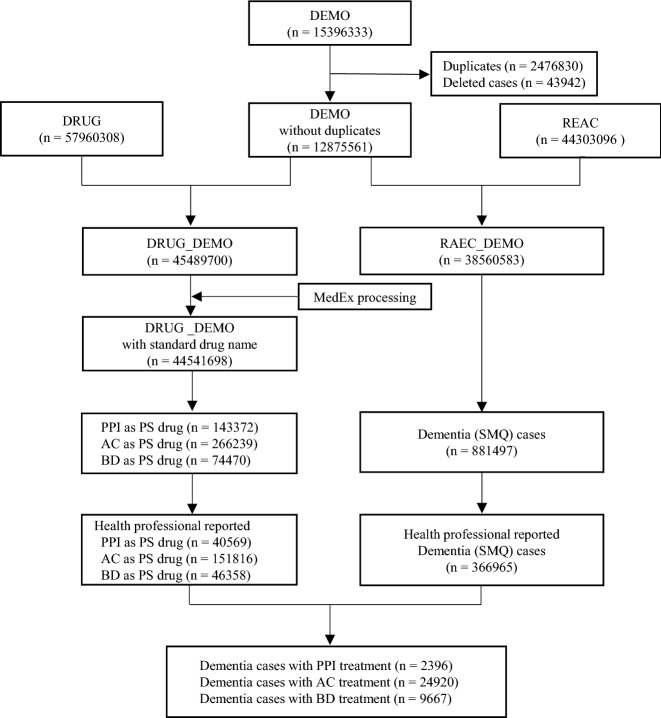
Flow chart of identifying dementia cases with target drug treatment reported by health professionals from FAERS database. *FAERS* FDA adverse events reporting system, *PPI* proton pump inhibitor, *AC* anticholinerigc drug, *BD* benzodiazepine drug, *SMQ* Standardised MedDRA Queries, *PS* primary suspected.

After data cleaning, we retrieved a total of 12,875,561 cases from January 2004 to December 2020, 6,074,285 of which were reported by health professionals. We finally identified 2396 PPI cases, as well as 24,920 anticholinerigc drug (AC) and 9667 benzodiazepine drug (BD) cases as positive controls, with dementia event reported by health professionals (Fig. 1). No case was identified for dexrabeprazole.

**Figure 2:**
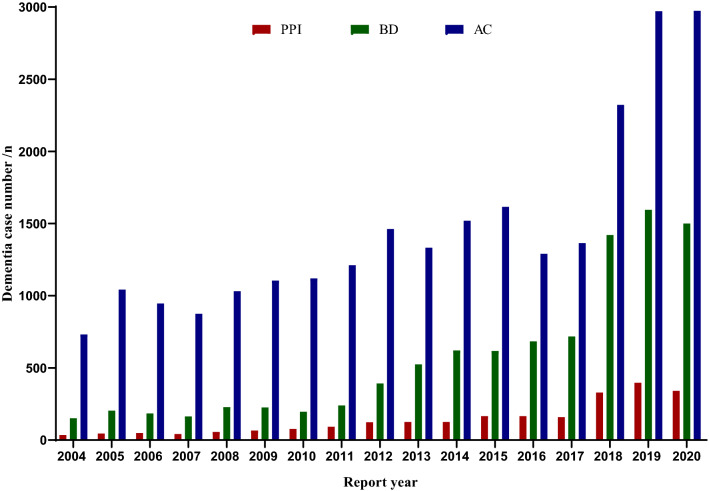
Annual reports of dementia cases with target drug treatment by health professionals in FAERS. *FAERS* FDA adverse events reporting system, *PPI* proton pump inhibitor, *BD* benzodiazepine drug, *AC* anticholinerigc drug.

The characteristic of PPI and positive control case was shown in Table 1. Among dementia cases reported with age, the highest proportion of PPI, AC and BD users with dementia event were below-65-year group. The ratio of female versus male dementia case was 1.40 for PPI, 1.04 for AC and 1.41 for BD, more female cases reported. The highest proportion of reporter occupation was other health professional for PPI (52.50%), physician for AC (46.00%) and BD (47.80%). The top one reporter country was Great Britain for PPI (26.70%), United States for AC (23.50%) and Italy for BD (33.20%). The number of dementia event case with PPI, AC, BD and other primary suspected drug therapy was almost increasing year by year (Fig. 2).

**Table 1:** Characteristics of target drug cases reported by health professionals in FAERS.

Characteristics	PPI (n/%)		AC (n/%)		BD (n/%)	
	Dementia case	Non-dementia case	Dementia case	Non-dementia case	Dementia case	Non-dementia case
Case	2395 (100.00)	38,162 (100.00)	24,913 (100.00)	126,873 (100.00)	9664 (100.00)	36,684 (100.00)
**Age group**						
< 65 years	988 (41.30)	14,790 (38.80)	14,211 (57.00)	70,549 (55.60)	5884 (60.90)	24,908 (67.90)
65 to 85 years	808 (33.70)	11,632 (30.50)	3606 (14.50)	14,710 (11.60)	1675 (17.30)	3702 (10.10)
≥ 85 years	264 (11.00)	2337 (6.10)	913 (3.70)	2427 (1.90)	463 (4.80)	699 (1.90)
Unknown	335 (14.00)	9403 (24.60)	6183 (24.80)	39,187 (30.90)	1642 (17.00)	7375 (20.10)
**Sex**						
Female	1300 (54.30)	18,654 (48.90)	11,785 (47.30)	56,299 (44.40)	5239 (54.20)	17,367 (47.30)
Male	926 (38.70)	14,315 (37.50)	11,354 (45.60)	56,990 (44.90)	3725 (38.50)	14,959 (40.80)
Unknown	169 (7.10)	5193 (13.60)	1774 (7.10)	13,584 (10.70)	700 (7.20)	4358 (11.90)
**Reporter occupation**						
Physician	818 (34.20)	14,881 (39.00)	11,465 (46.00)	58,213 (45.90)	4624 (47.80)	17,118 (46.70)
Pharmacist	320 (13.40)	5151 (13.50)	3290 (13.20)	20,301 (16.00)	1350 (14.00)	7365 (20.10)
Other health professional	1257 (52.50)	18,130 (47.50)	10,158 (40.80)	48,359 (38.10)	3690 (38.20)	12,201 (33.30)
**Top 5 reporter country (sort by PPI)**						
Great Britain	639 (26.70)	5002 (13.10)	3851 (15.50)	17,523 (13.80)	365 (3.80)	1214 (3.30)
France	394 (16.50)	6990 (18.30)	1512 (6.10)	6698 (5.30)	1434 (14.80)	4778 (13.00)
United States	346 (14.40)	11,733 (30.70)	5843 (23.50)	49,932 (39.40)	2199 (22.80)	19,141 (52.20)
Italy	189 (7.90)	1539 (4.00)	2936 (11.80)	3508 (2.80)	3212 (33.20)	2535 (6.90)
Canada	183 (7.60)	1337 (3.50)	1242 (5.00)	6975 (5.50)	263 (2.70)	1055 (2.90)

*FAERS* FDA adverse events reporting system, *PPI* proton pump inhibitor, *AC* anticholinerigc drug, *BD* benzodiazepine drug.

### Signal detection

**Figure 3:**
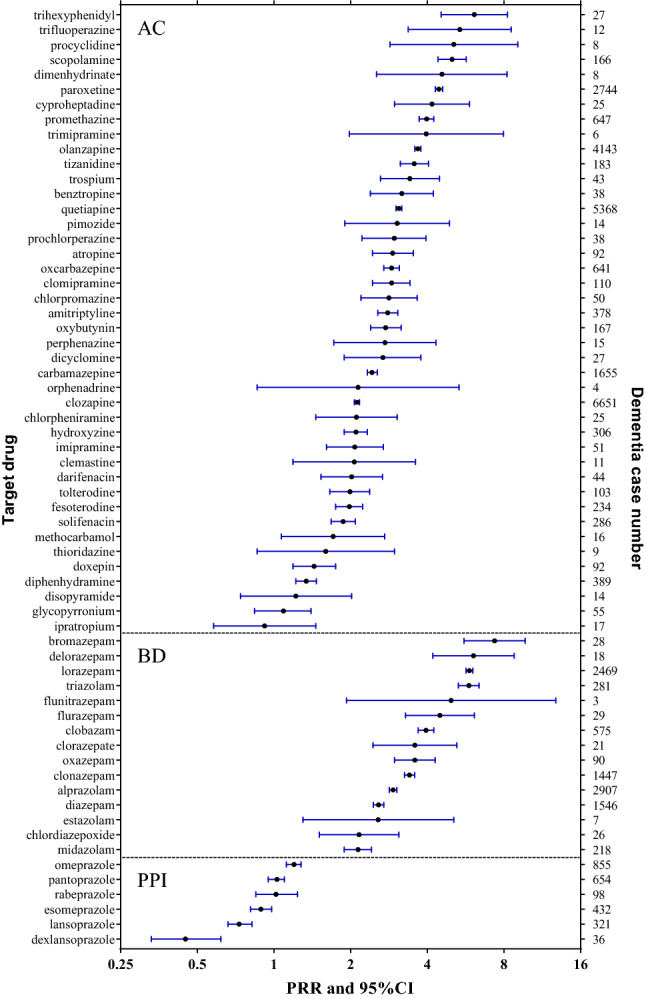
PRR and 95%CI of each single drug associated dementia reported by health professionals in FAERS (Dementia case number ≥ 3 ). *AC* anticholinerigc drug, *BD* benzodiazepine drug, *PPI* proton pump inhibitor, *FAERS* FDA adverse events reporting system, *PRR* proportional reporting ratio, *95%CI* 95% confidence interval.

We first conducted signal detection based on all indication population, detected no significant association between PPI use and dementia event (Table 2), PRR = 0.98, 95%CI 0.94–1.02, IC = −0.03, 95%CI − 0.17 to 0.10, however, significant signal was detected in both AC (PRR = 2.84, 95%CI 2.81–2.88, IC = 1.44, 95%CI 1.40–1.49) and BD (PRR = 3.52, 95%CI 3.46–3.58, IC = 1.79, 95%CI 1.71–1.86). We also conducted signal detection in individual PPI agents, detected no significant signals in all the six PPI agents as well. Signal detection of each single drug associated dementia was shown in Fig. 3.

**Table 2:** Signal detection for drug associated dementia reported by health professionals in FAERS.

Drugs	Dementia cases (n/%)	All AE cases (N)	PRR	95%CI for PRR	IC	95%CI for IC
**All indication cases**						
PPI	2396 (5.91)	40,569	0.98	0.94 to 1.02	− 0.03	− 0.17 to 0.10
AC	24,920 (16.41)	151,816	2.84	2.81 to 2.88	1.44	1.40 to 1.49
BD	9667 (20.85)	46,358	3.52	3.46 to 3.58	1.79	1.71 to 1.86
**GERD indication cases**						
All PPI	355 (3.92)	9053	0.65	0.59 to 0.72	− 0.62	− 0.97 to − 0.27
Dexlansoprazole	17 (2.40)	707	0.40	0.25 to 0.64	− 1.33	− 2.84 to 0.27
Esomeprazole	80 (5.29)	1511	0.88	0.71 to 1.08	− 0.19	− 0.93 to 0.55
Lansoprazole	53 (2.47)	2148	0.41	0.31 to 0.53	− 1.29	− 2.17 to − 0.38
Omeprazole	89 (4.12)	2161	0.68	0.56 to 0.84	− 0.55	− 1.25 to 0.15
Pantoprazole	88 (4.11)	2139	0.68	0.55 to 0.84	− 0.55	− 1.25 to 0.15
Rabeprazole	28 (7.24)	387	1.20	0.84 to 1.71	0.26	− 1.01 to 1.50

*PPI* proton pump inhibitor, *AC* anticholinerigc drug, *BD* benzodiazepine drug, *FAERS* FDA adverse events reporting system, *AE* adverse event, *PRR* proportional reporting ratio, *IC* information element, *95%CI* 95% confidence interval, *GERD* gastroesophageal reflux disease.

We then conducted PPI signal detection based on cases with indication of GERD, 355 dementia cases were gathered out of 9053 PPI users with GERD indication reported by health professionals in FAERS. However, no significant signal between PPI treatment and dementia event was detected, PRR = 0.65, 95%CI 0.59–0.72, IC = −0.62, 95%CI − 0.97 to − 0.27 (Table 2).

We further performed sensitivity analysis of signal detection for the association between PPI use and dementia event in three independent methods (Supplementary Table S1). The first way, we excluded cases with anti-dementia drugs as co-therapy from the previously included cases (Supplementary Table S1-1). The second way, we excluded cases with age blow 65 years from the previously included cases (Supplementary Table S1-2). The third way, we use SMQ narrow searching to re-identify health professional reported cases (Supplementary Table S1-3). All the three methods were done in both PPI case and the background case simultaneously, detected no significant association between PPI use and dementia event, indicating robust outcome.

### Time event comparison

We estimated the time interval from PPI use to adverse event onset, comparing dementia event between long- and short-time interval groups. 754 dementia cases were identified out of 11,033 PPI users with time interval data reported by health professionals in FAERS. We divided different long- and short-time interval with 3 years, 5 year and 10 years. However, no significant difference was found between each long- and short-term groups (Table 3). The long-term versus short-term OR value (95%CI) of the 3 years, 5 year and 10 years comparison were 0.70 (0.48–1.02), 0.72 (0.45–1.15) and 1.65 (0.75–3.63), indicating long term PPI use did not increasing dementia event.

**Table 3:** Comparison of dementia event between long-term and short-term proton pump inhibitors treatment cases.

Groups	Time interval	Dementia cases (n/%)	All AE cases (N)	OR		Chi-square test	
				Value	95%CI	χ^2^	*P* value
Groups1	> 3 years	30 (4.99)	601	0.70	0.48 to 1.02	3.389	0.066
	≤ 3 years	724 (6.94)	10,432				
Groups2	> 5 years	19 (5.05)	376	0.72	0.45 to 1.15	1.939	0.164
	≤ 5 years	735 (6.90)	10,657				
Groups3	> 10 years	7 (10.77)	65	1.65	0.75 to 3.63	1.029	0.310
	≤ 10 years	747 (6.81)	10,968				

*AE* adverse event, *OR* odds ratio, *95%CI* 95% confidence interval.

### PPI dosage

The dosage analysis found no difference of daily dosage for each PPI agent between dementia group and non-dementia group. Moreover, the median (IQR) daily dose of each PPI agent was within the range recommended by drug label (Table 4).

**Table 4:** Daily dosage of PPI agents reported by health professionals in FAERS.

Drug name	Dementia case			Non-dementia case			WHO DDD /mg	Daily dose recommended by drug label /mg
	Case with dose data /n	Dose (median) /mg	Dose (IQR) /mg	Case with dose data /n	Dose (median) /mg	Dose (IQR) /mg		
Dexlansoprazole	30	60	60–60	646	60	60–60	30	30–60
Esomeprazole	162	40	20–40	2530	40	20–40	30	20–40
Lansoprazole	151	30	15–30	2795	30	15–30	30	15–60
Omeprazole	312	20	20–40	3319	20	20–40	20	20–60
Pantoprazole	295	40	20–40	3832	40	20–40	40	40
Rabeprazole	34	20	10–20	522	20	10–20	20	20–60

*PPIs* proton pump inhibitors, *DDD* defined daily dose, *IQR* interquartile range.

## Discussion

The current study investigated the association between six PPI agents and dementia event, compared different time interval of PPI treatment and dementia event. The results indicated no association between dementia event and PPI agents, including dexlansoprazole, esomeprazole, lansoprazole, omeprazole, pantoprazole and rabeprazole. To the best of our knowledge, this was the first pharmacovigilance study concerned the association between PPI use and dementia event based on FAERS database.

With the widespread use of PPI agents, PPI-associated adverse event had caught health professionals’ attention, as well as the public and the media. In FAERS database, more than half of the PPI adverse event cases were reported by non-health professionals. To reduce the influence of non-health professionals, we only included cases reported by health professionals. However, the proportion of case reported by physician and pharmacist was less than a half for PPI, smaller than AC and BD. Moreover, the number of dementia case treated by PPI was increasing by years, especially from the year of 2018. The suddenly increased case number might be caused by the suddenly increased annual FAERS reports in 2018 and concerns of PPI safety in recent years. The risk of stimulated reporting, both by clinical evidence and media influence, could not be ruled out.

Based on the β-amyloid enhancement^8^, vitamin B12 deficiency phenomena^9^ and the widespread PPI use, the association between dementia event and PPI use had become a hot topic. Professor Akter et al. first revealed five different PPI agents had varying degrees of influence on different cognitive domains associated with dementia based on the Cambridge Neuropsychological Test Automated Battery (CANTAB) software test^10^. Professor Haenisch et al. conducted the first epidemiological investigation, indicated PPI might have an impact on dementia based on the German Study on Aging, Cognition and Dementia in Primary Care Patients (AgeCoDe)^11^. Then, professor Gomm et al. conducted the first prospective cohort study, revealed regular PPI treatment had a significantly increased risk of dementia using data derived from the largest German statutory health insurer, Allgemeine Ortskrankenkassen (AOK)^12^, which had been hotly commented.

However, conflicting results had been gradually published. Professor Lochhead et al. conducted a nationwide prospective cohort study and divided PPI users into four groups based on duration of PPI treatment, revealed a modest association between duration of PPI use and cognitive function, however, Lochhead stated that the result could not support PPI use increased dementia event^31^. Professor Taipale et al. finished a nationwide nested case–control study which set a lag window of different duration, found PPI use was not associated with risk of Alzheimer's disease with a 3-year lag window^32^. Professor Gray et al. reported a prospective cohort study and found no association between PPI exposure and dementia event after a mean follow-up of 7.5 years^20^. Professor Cooksey et al. conducted a large population-based study based on electronic health-data from the Secure Anonymised Information Linkage (SAIL) Databank from 1999 to 2015, could not confirm an association between PPI use and dementia event^17^. The current study based the FAERS big data, indicated no significant signal between PPI use and dementia event. Even compared in different time duration, no significant difference of dementia event was found between long- and short-term groups.

Our study revealed no association between PPI use and dementia event based on the FAERS real world big data, however, certain limitations existed. FAERS is a spontaneous reporting system, voluntary and opened to health professional as well as the public, so under-reporting, over-reporting or missing data was inevitable^33^. The time event comparison only included limited cases with time data reported. Although non-health professionals’ reports excluded, the risk of stimulated reporting could not be eliminated.

In summary, the current study revealed no association between six PPI agents and dementia event based on the FAERS data mining. Our findings suggested that dementia event might not be considered as a factor in discontinuing PPI treatment.

## Materials and methods

### Data source

We downloaded FAERS data from January 2004 to December 2020 in the FAERS Quarterly Data Extract Files website^34^. FAERS data was processed anonymously, no ethical review was required.

The FAERS datasets consisted of seven data tables as follow: “DEMO” table for patient demographic and administrative information, “DRUG” table for the drug information, “REAC” table for adverse events information, “OUTC” table for patient outcomes information, “RPSR” table for report sources information, “THER” table for drug therapy start and end dates information and “INDI” table for the indications for drug use. We managed FAERS data in local by Microsoft SQL server 2017 software.

We first removed duplicated cases from the original data as the FDA recommended. We removed the same records from “DEMO” table and left one, then deleted the earliest FDA_DT when the CASEIDs were the same and removed the lower PRIMARYID when the CASEID and FDA_DT were the same. We further removed cases listed in the FAERS deleted files. In the current study, we only included cases reported by health professionals, including physicians, pharmacists and other health professionals, for both target drug data and background data.

### Target drug identification

In “DRUG” table, drugs could be documented in various forms, such as generic names, brand names, synonymous names or their abbreviations. We used the MedEx software (MedEx UIMA 1.3.8, Vanderbilt university, US) to standardize different names of the same drug into the “generic name”^35,36^.

We tried to identify seven single component PPI agents with the WHO Anatomical Therapeutic Chemical (ATC) code of A02BC from local FAERS database. The seven PPI agents (ATC code) included omeprazole (A02BC01), pantoprazole (A02BC02), lansoprazole (A02BC03), rabeprazole (A02BC04), esomeprazole (A02BC05), dexlansoprazole (A02BC06) and dexrabeprazole (A02BC07). We restricted the drug role as Primary Suspected (PS) drug.

We also identified anticholinerigc drugs (AC) and benzodiazepine drugs (BD) as positive controls. We included 56 AC drugs following the Coupland et al. study^37^ and 30 BD drugs following three BD data mining studies^38–40^, detailed in Supplementary Table S2.

### Dementia event identification

According to Medical Dictionary for Regularly Activities (MedDRA) and Standardised MedDRA Queries (SMQs) version 23.1. We identified dementia cases in “REAC” table using SMQ (code: 20,000,073) broad searching, including 105 Preferred Terms (PTs). For cases reported more than one PT of the same SMQ, we removed duplicate records and kept one. The PTs details could be found in Supplementary Table S3.

### Data mining

We gathered the characteristics of dementia case with PPI, AC and BD, including age and sex, reporter and report country, annual case reported, indications and daily dosage.

We employed both proportional reporting ratio (PRR, a frequency method) and information component (IC, a Bayesian method) to detect signals of dementia event relevant to PPI, as well as AC and BD positive controls. The calculation method of PRR, IC and the 95% confidence interval (95% CI) were shown in Supplementary Table S4. A significant signal was defined as both PRR and IC signal detected. The PRR signal criteria was case number ≥ 3, PRR ≥ 2 and χ^2^ ≥ 4^41^. The IC signal criteria was IC > 0 and the lower limit of 95% CI > 0^28^.

We further calculated signal between PPI use and dementia event in GERD cases who might receive long-term PPI treatment.

### Statistical analysis

We estimated the time interval from PPI use to adverse event reported in all PPI (PS) cases reported by health professionals in FAERS. We unified the time format as yyyy-mm-dd. The time interval was calculated using event date (EVENT_DT) minus drug start date (START_DT). To make the calculation more accurately, we excluded cases not in the period of 2004–2020, cases without year or month data in either EVENT_DT or START_DT field, and cases with earlier event date than drug start date. For the long- and short-duration comparison, we calculated the odds ratio (OR) using formula in Supplementary Table S5 and performed Pearson’s chi-squared test using SPSS software. *P* value less than 0.05 indicated significant difference.

The statistical analyses were conducted by Microsoft Excel version 2013 (Microsoft corporation, Redmond, Washington, USA), SPSS version 25.0 (IBM corporation, Armonk, New York, USA) and GraphPad prism version 8.0.2 (GraphPad Software, San Diego, California, USA).

## Supplementary Information

Supplementary Information.
