# Supplementary material for: A pharmacovigilance study of association between proton pump inhibitor and dementia event based on FDA adverse event reporting system data
Source: Sci Rep. 2021 May 21;11:10709. doi: 10.1038/s41598-021-90108-7 (PMC8139970; doi:10.1038/s41598-021-90108-7)
Supplement: Supplementary file 1 — Supplementary Information. [file 41598_2021_90108_MOESM1_ESM.docx]

**A pharmacovigilance study of association between proton pump inhibitor and dementia event based on FDA adverse event reporting system data**

Bin Wu^1^, Qiaozhi Hu^1^, Fangyuan Tian^1^, Fengbo Wu ^1^, Yuwen Li ^1^, Ting Xu *^1, 2^

**Supplementary Table S1. Sensitivity analysis of signal detection for the association between PPI and dementia event reported in FAERS**

Supplementary Table S1-1. Dementia cases (SMQ border searching) with anti-dementia drugs therapy case excluded.

| **Drugs** | **Dementia cases (n/%)** | **All AE cases (N)** | **PRR** | **95%CI for PRR** | **IC** | **95%CI for IC** |
| --- | --- | --- | --- | --- | --- | --- |
| AC | 24439(16.26) | 150328 | 2.87 | 2.84 ~ 2.91 | 1.46 | 1.41 ~ 1.50 |
| BD | 9563(20.75) | 46095 | 3.57 | 3.51 ~ 3.64 | 1.81 | 1.73 ~ 1.88 |
| PPI | 2344(5.82) | 40243 | 0.98 | 0.94 ~ 1.02 | -0.02 | -0.16 ~ 0.11 |
| Dexlansoprazole | 36(2.73) | 1318 | 0.46 | 0.33 ~ 0.64 | -1.12 | -2.18 ~ -0.01 |
| Esomeprazole | 425(5.34) | 7952 | 0.90 | 0.82 ~ 0.99 | -0.15 | -0.47 ~ 0.18 |
| Lansoprazole | 316(4.40) | 7185 | 0.74 | 0.67 ~ 0.83 | -0.43 | -0.80 ~ -0.06 |
| Omeprazole | 826(7.04) | 11740 | 1.19 | 1.11 ~ 1.27 | 0.25 | 0.01 ~ 0.48 |
| Pantoprazole | 646(6.16) | 10485 | 1.04 | 0.96 ~ 1.12 | 0.06 | -0.21 ~ 0.32 |
| Rabeprazole | 95(6.08) | 1563 | 1.03 | 0.84 ~ 1.25 | 0.04 | -0.65 ~ 0.72 |

Supplementary Table S1-2. Dementia cases (SMQ border searching) with young case (＜65 years) excluded.

| **Drugs** | **Dementia cases (n/%)** | **All AE cases (N)** | **PRR** | **95%CI for PRR** | **IC** | **95%CI for IC** |
| --- | --- | --- | --- | --- | --- | --- |
| AC | 4518(22.12) | 20426 | 3.46 | 3.37 ~ 3.55 | 1.74 | 1.63 ~ 1.85 |
| BD | 2138(34.71) | 6159 | 5.35 | 5.16 ~ 5.54 | 2.39 | 2.23 ~ 2.55 |
| PPI | 1072(7.50) | 14297 | 1.14 | 1.07 ~ 1.20 | 0.18 | -0.03 ~ 0.39 |
| Dexlansoprazole | 12(5.91) | 203 | 0.89 | 0.52 ~ 1.55 | -0.16 | -2.02 ~ 1.71 |
| Esomeprazole | 174(5.78) | 3012 | 0.87 | 0.76 ~ 1.01 | -0.19 | -0.70 ~ 0.31 |
| Lansoprazole | 168(7.74) | 2170 | 1.17 | 1.01 ~ 1.35 | 0.23 | -0.29 ~ 0.75 |
| Omeprazole | 384(8.46) | 4537 | 1.28 | 1.16 ~ 1.41 | 0.36 | 0.01 ~ 0.70 |
| Pantoprazole | 281(7.55) | 3723 | 1.14 | 1.02 ~ 1.28 | 0.19 | -0.21 ~ 0.59 |
| Rabeprazole | 53(8.13) | 652 | 1.23 | 0.95 ~ 1.59 | 0.30 | -0.63 ~ 1.21 |

Supplementary Table S1-3. Dementia cases (SMQ narrow searching).

| **Drugs** | **Dementia cases (n/%)** | **All AE cases (N)** | **PRR** | **95%CI for PRR** | **IC** | **95%CI for IC** |
| --- | --- | --- | --- | --- | --- | --- |
| AC | 646(0.43) | 151816 | 2.71 | 2.51 ~ 2.94 | 1.38 | 1.11 ~ 1.64 |
| BD | 75(0.16) | 46358 | 0.99 | 0.79 ~ 1.24 | -0.02 | -0.77 ~ 0.73 |
| PPI | 49(0.12) | 40569 | 0.74 | 0.56 ~ 0.98 | -0.44 | -1.35 ~ 0.49 |
| Dexlansoprazole | 0(0.00) | 1324 | - | - | - | - |
| Esomeprazole | 24(0.30) | 8034 | 1.83 | 1.23 ~ 2.73 | 0.87 | -0.48 ~ 2.13 |
| Lansoprazole | 4(0.06) | 7240 | 0.34 | 0.13 ~ 0.90 | -1.57 | -4.27 ~ 1.55 |
| Omeprazole | 11(0.09) | 11833 | 0.57 | 0.31 ~ 1.03 | -0.81 | -2.64 ~ 1.12 |
| Pantoprazole | 8(0.08) | 10555 | 0.46 | 0.23 ~ 0.93 | -1.11 | -3.19 ~ 1.15 |
| Rabeprazole | 2(0.13) | 1583 | 0.77 | 0.19 ~ 3.09 | -0.37 | -4.02 ~ 3.50 |

AC: anticholinerigc drug; BD: benzodiazepine drug; PPI: proton pump inhibitor; AE: adverse event; PRR: proportional reporting ratio; IC: information element; 95%CI: 95% confidence interval.

**Supplementary Table S2. Included and excluded drugs for FAERS data mining.**

| **No.** | **Drug type** | **Drug name** | **No.** | **Drug type** | **Drug name** | **No.** | **Drug type** | **Drug name** |
| --- | --- | --- | --- | --- | --- | --- | --- | --- |
| 1 | **PPI** | Omeprazole | 35 | **AC** | Procyclidine | 69 | **BD** | Clonazepam |
| 2 | **PPI** | Pantoprazole | 36 | **AC** | Trihexyphenidyl | 70 | **BD** | Clorazepate |
| 3 | **PPI** | Lansoprazole | 37 | **AC** | Chlorpromazine | 71 | **BD** | Clotiazepam |
| 4 | **PPI** | Rabeprazole | 38 | **AC** | Clozapine | 72 | **BD** | Cloxazolam |
| 5 | **PPI** | Esomeprazole | 39 | **AC** | Methotrimeprazine | 73 | **BD** | Diazepam |
| 6 | **PPI** | Dexlansoprazole | 40 | **AC** | Olanzapine | 74 | **BD** | Estazolam |
| 7 | **PPI** | Dexrabeprazole | 41 | **AC** | Pericyazine | 75 | **BD** | Ethylloflazepate |
| 8 | **AC** | Disopyramide | 42 | **AC** | Perphenazine | 76 | **BD** | Etizolam |
| 9 | **AC** | Azatadine | 43 | **AC** | Pimozide | 77 | **BD** | Fludiazepam |
| 10 | **AC** | Brompheniramine | 44 | **AC** | Quetiapine | 78 | **BD** | Flunitrazepam |
| 11 | **AC** | Chlorpheniramine | 45 | **AC** | Thioridazine | 79 | **BD** | Flurazepam |
| 12 | **AC** | Clemastine | 46 | **AC** | Trifluoperazine | 80 | **BD** | Flutazolam |
| 13 | **AC** | Cyproheptadine | 47 | **AC** | Darifenacin | 81 | **BD** | Flutoprazepam |
| 14 | **AC** | Diphenhydramine | 48 | **AC** | Fesoterodine | 82 | **BD** | Loflazepate |
| 15 | **AC** | Hydroxyzine | 49 | **AC** | Flavoxate | 83 | **BD** | Lorazepam |
| 16 | **AC** | Alimemazine | 50 | **AC** | Oxybutynin | 84 | **BD** | Medazepam |
| 17 | **AC** | Amitriptyline | 51 | **AC** | Propiverine | 85 | **BD** | Mexazolam |
| 18 | **AC** | Clomipramine | 52 | **AC** | Solifenacin | 86 | **BD** | Midazolam |
| 19 | **AC** | Dosulepin | 53 | **AC** | Tolterodine | 87 | **BD** | Nimetazepam |
| 20 | **AC** | Doxepin | 54 | **AC** | Trospium | 88 | **BD** | Oxazepam |
| 21 | **AC** | Imipramine | 55 | **AC** | Methocarbamol | 89 | **BD** | Oxazolam |
| 22 | **AC** | Lofepramine | 56 | **AC** | Tizanidine | 90 | **BD** | Pinazepam |
| 23 | **AC** | Nortiptyline | 57 | **AC** | Alverine | 91 | **BD** | Prazepam |
| 24 | **AC** | Paroxetine | 58 | **AC** | Atropine | 92 | **BD** | Tofisopam |
| 25 | **AC** | Trimipramine | 59 | **AC** | Dicyclomine | 93 | **BD** | Triazolam |
| 26 | **AC** | Carbamazepine | 60 | **AC** | Propantheline | 94 | **ATD** | Tacrine |
| 27 | **AC** | Oxcarbazepine | 61 | **AC** | Scopolamine | 95 | **ATD** | Donepezil |
| 28 | **AC** | Cyclizine | 62 | **AC** | Glycopyrronium | 96 | **ATD** | Rivastigmine |
| 29 | **AC** | Dimenhydrinate | 63 | **AC** | Ipratropium | 97 | **ATD** | Galantamine |
| 30 | **AC** | Prochlorperazine | 64 | **BD** | Alprazolam | 98 | **ATD** | Ipidacrine |
| 31 | **AC** | Promethazine | 65 | **BD** | Bromazepam | 99 | **ATD** | Memantine |
| 32 | **AC** | Promazine | 66 | **BD** | Brotizolam | 100 | **ATD** | Ginkgo Folium |
| 33 | **AC** | Benztropine | 67 | **BD** | Chlordiazepoxide |  |  |  |
| 34 | **AC** | Orphenadrine | 68 | **BD** | Clobazam |  |  |  |

Included drugs for signal detection: PPI: proton pump inhibitor; AC: anticholinerigc drug; BD: benzodiazepine drug. Excluded drugs for sensitivity analysis: ATD: Anti-dementia drugs.

**Supplementary Table S3. Preferred terms for identifying of dementia case by SMQ (code: 20000073) searching.**

| **No.** | **PT code** | **Preferred term (PT)** | **Term scope** |
| --- | --- | --- | --- |
| 1 | 10011384 | Creutzfeldt-Jakob disease | 2 |
| 2 | 10012267 | Dementia | 2 |
| 3 | 10012271 | Dementia Alzheimer's type | 2 |
| 4 | 10012293 | Dementia of the Alzheimer's type, uncomplicated | 2 |
| 5 | 10012294 | Dementia of the Alzheimer's type, with delirium | 2 |
| 6 | 10012295 | Dementia of the Alzheimer's type, with delusions | 2 |
| 7 | 10012296 | Dementia of the Alzheimer's type, with depressed mood | 2 |
| 8 | 10036631 | Presenile dementia | 2 |
| 9 | 10036813 | Progressive supranuclear palsy | 2 |
| 10 | 10039966 | Senile dementia | 2 |
| 11 | 10057678 | Vascular dementia | 2 |
| 12 | 10063629 | Hippocampal sclerosis | 2 |
| 13 | 10064199 | Variant Creutzfeldt-Jakob disease | 2 |
| 14 | 10065424 | Mini mental status examination abnormal | 2 |
| 15 | 10067889 | Dementia with Lewy bodies | 2 |
| 16 | 10068968 | Frontotemporal dementia | 2 |
| 17 | 10070997 | Korsakoff's syndrome | 2 |
| 18 | 10075174 | Mixed dementia | 2 |
| 19 | 10077237 | Scatolia | 2 |
| 20 | 10078036 | Early onset familial Alzheimer's disease | 2 |
| 21 | 10078208 | Corticobasal degeneration | 2 |
| 22 | 10079301 | Prion disease | 2 |
| 23 | 10079991 | Clinical dementia rating scale score abnormal | 2 |
| 24 | 10081973 | Hippocampal atrophy | 2 |
| 25 | 10001488 | Aggression | 1 |
| 26 | 10001497 | Agitation | 1 |
| 27 | 10001949 | Amnesia | 1 |
| 28 | 10002711 | Anterograde amnesia | 1 |
| 29 | 10002942 | Apathy | 1 |
| 30 | 10002948 | Aphasia | 1 |
| 31 | 10003062 | Apraxia | 1 |
| 32 | 10004207 | Behaviour disorder | 1 |
| 33 | 10008096 | Cerebral atrophy | 1 |
| 34 | 10008398 | Change in sustained attention | 1 |
| 35 | 10010305 | Confusional state | 1 |
| 36 | 10012218 | Delirium | 1 |
| 37 | 10012239 | Delusion | 1 |
| 38 | 10012251 | Delusional disorder, jealous type | 1 |
| 39 | 10012255 | Delusional disorder, unspecified type | 1 |
| 40 | 10013142 | Disinhibition | 1 |
| 41 | 10013395 | Disorientation | 1 |
| 42 | 10016322 | Feeling abnormal | 1 |
| 43 | 10016759 | Flat affect | 1 |
| 44 | 10019063 | Hallucination | 1 |
| 45 | 10019070 | Hallucination, auditory | 1 |
| 46 | 10019075 | Hallucination, visual | 1 |
| 47 | 10019079 | Hallucinations, mixed | 1 |
| 48 | 10020400 | Hostility | 1 |
| 49 | 10021030 | Hypomania | 1 |
| 50 | 10021403 | Illusion | 1 |
| 51 | 10021588 | Inappropriate affect | 1 |
| 52 | 10022035 | Initial insomnia | 1 |
| 53 | 10023236 | Judgement impaired | 1 |
| 54 | 10024092 | Learning disability | 1 |
| 55 | 10027175 | Memory impairment | 1 |
| 56 | 10027374 | Mental impairment | 1 |
| 57 | 10027940 | Mood altered | 1 |
| 58 | 10027951 | Mood swings | 1 |
| 59 | 10027977 | Morose | 1 |
| 60 | 10028899 | Negativism | 1 |
| 61 | 10034719 | Personality change | 1 |
| 62 | 10037211 | Psychomotor hyperactivity | 1 |
| 63 | 10037249 | Psychotic behaviour | 1 |
| 64 | 10038743 | Restlessness | 1 |
| 65 | 10040045 | Separation anxiety disorder | 1 |
| 66 | 10041243 | Social avoidant behaviour | 1 |
| 67 | 10041347 | Somnambulism | 1 |
| 68 | 10041349 | Somnolence | 1 |
| 69 | 10041466 | Speech disorder | 1 |
| 70 | 10042635 | Suspiciousness | 1 |
| 71 | 10043431 | Thinking abnormal | 1 |
| 72 | 10044380 | Transient global amnesia | 1 |
| 73 | 10048294 | Mental status changes | 1 |
| 74 | 10048663 | Agnosia | 1 |
| 75 | 10050013 | Abulia | 1 |
| 76 | 10052236 | Cerebral atrophy congenital | 1 |
| 77 | 10052393 | Borderline mental impairment | 1 |
| 78 | 10054196 | Affect lability | 1 |
| 79 | 10057224 | Irritability postvaccinal | 1 |
| 80 | 10057668 | Cognitive disorder | 1 |
| 81 | 10058709 | Sopor | 1 |
| 82 | 10060082 | Intelligence test abnormal | 1 |
| 83 | 10061108 | Disturbance in social behaviour | 1 |
| 84 | 10061265 | Learning disorder | 1 |
| 85 | 10061284 | Mental disorder | 1 |
| 86 | 10061422 | Abnormal behaviour | 1 |
| 87 | 10061423 | Amnestic disorder | 1 |
| 88 | 10061920 | Psychotic disorder | 1 |
| 89 | 10065426 | Neuropsychological test abnormal | 1 |
| 90 | 10068855 | Symbolic dysfunction | 1 |
| 91 | 10070240 | Sexually inappropriate behaviour | 1 |
| 92 | 10070246 | Executive dysfunction | 1 |
| 93 | 10070920 | Visual cortex atrophy | 1 |
| 94 | 10071176 | Impaired reasoning | 1 |
| 95 | 10074570 | Vascular cognitive impairment | 1 |
| 96 | 10074616 | Prodromal Alzheimer's disease | 1 |
| 97 | 10077174 | Neuritic plaques | 1 |
| 98 | 10077244 | Defiant behaviour | 1 |
| 99 | 10078497 | Neuropsychiatric symptoms | 1 |
| 100 | 10079487 | Loss of personal independence in daily activities | 1 |
| 101 | 10080880 | Visuospatial deficit | 1 |
| 102 | 10081268 | Primary progressive aphasia | 1 |
| 103 | 10082331 | Aberrant motor behaviour | 1 |
| 104 | 10084030 | Delusion of theft | 1 |
| 105 | 10084569 | Patient elopement | 1 |

Where term scope 2 means SMQ narrow searching, term scope 1 plus 2 means broad searching.

**Supplementary Table S4. Two-by-two contingency table for PRR and IC analyses**

| **Drugs** | **Dementia event cases** | **All other adverse event cases** |
| --- | --- | --- |
| Target drug | a | b |
| All other drugs | c | d |
| PRR = $\frac{a/(a+b)}{\text{c}/\text{(}c+d)}$  95%CI for PRR = $e^{\ln(PRR)\pm1.96\sqrt{(\frac{1}{a}-\frac{1}{a+b}+\frac{1}{c}-\frac{1}{c+d})}}$ | | |
| IC = log_2_$\frac{a(a+b+c+d)}{\text{(a+b)(a}+c)}$  E(IC) = log_2_$\frac{(a+\gamma11)(N+\alpha)(N+\beta)}{\text{(N+γ)(a}+b+\alpha1)(a+c+\beta1)}$  V(IC) ≈ ($\frac{1}{\text{log2}}$)^2^[$\frac{N-a+\gamma-\gamma11)}{\text{(a+γ11)(1}+N+\gamma)}+\frac{N-a-b+\alpha-\alpha1)}{\text{(a+b+α1)(1}+N+\alpha)}+\frac{N-a-c+\beta-\beta1)}{\text{(a+c+β1)(1}+N+\beta)}$]  γ = γ_11_$\frac{(N+\alpha)(N+\beta)}{\text{(a+b+α1)(a}+c+\beta1)}$  95%CI for IC = E(IC) ± 1.96$\sqrt{V(IC)}$  Where α=α_1_+α_2_, β=β_1_+β_2_, N=a+b+c+d, and the value of α_1_, α_2_, β_1_, β_2_ and γ_11_ were defined as 1. | | |

PRR: proportional reporting ratio; IC: information component; 95%CI: 95% confidence interval.

**Supplementary Table S5. Fourfold table for OR analyses**

|  | **Dementia event cases** | **Non-dementia event cases** |
| --- | --- | --- |
| Long-term PPI use | a | b |
| Short-term PPI use | c | d |
| OR = $\frac{a/b}{\text{c}/d}$  95%CI for OR = $e^{\text{ln(OR)}\pm\text{1.96}\sqrt{(\frac{1}{a}+\frac{1}{b}+\frac{1}{c}+\frac{1}{d})}}$ | | |
